# Association between Genetic Polymorphism of GSTP1 and Toxicities in Patients Receiving Platinum-Based Chemotherapy: A Systematic Review and Meta-Analysis

**DOI:** 10.3390/ph15040439

**Published:** 2022-04-01

**Authors:** Woorim Kim, Young-Ah Cho, Dong-Chul Kim, Kyung-Eun Lee

**Affiliations:** 1 College of Pharmacy, Chungbuk National University, Cheongju 28160, Korea; wanppa@hotmail.com; 2 College of Pharmacy, Gyeongsang National University, Jinju 52828, Korea; cyapham@hanmail.net; 3 Department of Pharmacy, The Prime Hospital, Jinju 52642, Korea; 4 Department of Pathology, Gyeongsang National University Hospital, Jinju 52727, Korea; 5 School of Medicine, Gyeongsang National University, Jinju 52828, Korea

**Keywords:** platinum, glutathione S-transferase pi 1, GSTP1, toxicity, meta-analysis

## Abstract

Platinum-based chemotherapy regimens have been proven to be effective in various cancers; however, considerable toxicities may develop and can even lead to treatment discontinuation. Diverse factors may influence adverse treatment events, with pharmacogenetic variations being one prime example. Polymorphisms within the glutathione S-transferase pi 1 (GSTP1) gene may especially alter enzyme activity and, consequently, various toxicities in patients receiving platinum-based chemotherapy. Due to a lack of consistency in the degree of elevated complication risk, we performed a systematic literature review and meta-analysis to determine the level of platinum-associated toxicity in patients with the GSTP1 rs1695 polymorphism. We conducted a systematic search for eligible studies published before January 2022 from PubMed, Web of Science, and EMBASE based on Preferred Reporting Items for Systematic Reviews and Meta-Analyses guidelines. Odds ratios (ORs) and 95% confidence intervals (CIs) were calculated to evaluate the strength of the association between the rs1695 polymorphism and various toxicities. Ten eligible studies met the inclusion criteria. The pooled ORs for hematological toxicity and neutropenia in the patients with the variant (G) allele were 1.7- and 2.6-times higher than those with the AA genotype (95% CI 1.06–2.73 and 1.07–6.35), respectively. In contrast, the rs1695 polymorphism resulted in a 44% reduced gastrointestinal toxicity compared to wild-type homozygotes. Our study found that the GSTP1 rs1695 polymorphism was significantly correlated with platinum-induced toxicities. The study also revealed that rs1695 expression exhibited tissue-specific patterns and thus yielded opposite effects in different tissues. A personalized chemotherapy treatment based on these polymorphisms may be considered for cancer patients in the future.

## 1. Introduction

For several decades, platinum-based cancer treatment has been a key component of chemotherapy regimens; it has been reported that nearly 50% of all cancer treatments involve platinum-based regimens [1]. Among platinum-based cancer drugs, cisplatin, oxaliplatin, and carboplatin are the top three most used platinum chemotherapy agents [2]. Cisplatin, the first FDA-approved platinum compound for chemotherapy, is the most commonly used drug in the treatment of several cancers, including testicular cancer, ovarian cancer, head and neck cancer, esophageal cancer, and lung cancer. Carboplatin is a second-generation platinum drug with activity equivalent to cisplatin, while oxaliplatin is a third-generation platinum drug with similar effectiveness and safety to cisplatin. Although platinum-based agents have been effective, a variety of adverse events have been consistently reported.

Glutathione S-transferase (GST) is a phase II enzyme that detoxifies substances, including platinum chemotherapy drugs [3]. GST Pi 1 (GSTP1) belongs to one of four major classes of GSTs and is associated with a sensitivity to platinum chemotherapy drugs [4]. It plays an important role in mediating the interaction between medications and glutathione [5]; hence, polymorphisms within the GSTP1 may result in changes in enzyme activity. Especially given that alterations in GST can result in decreased platinum detoxification, causing various toxicities [3]. Since polymorphisms in GSTP1 may cause GST abnormalities, genetic screening can be proposed in patients receiving platinum-based chemotherapy.

GSTP1 I105V (rs1695 A > G) is a single nucleotide polymorphism (SNP) located within the substrate-binding domain, resulting in an amino-acid substitution [6]. It is known that this polymorphism is associated with enzyme activity and clinical outcomes of platinum-based regimens [7,8]. GSTP1 rs1695 was widely studied in candidate gene association investigations, but results were inconsistent [9,10]; in one study, patients with rs1695 variants had decreased neurotoxicity [11], whereas, in another, opposite results were presented [3]. In this context, identifying the association between polymorphisms and platinum-related toxicities in a larger number of subjects would be beneficial for individualized chemotherapy. Hence, this study aims to investigate the potential link between the rs1695 polymorphism and platinum-induced toxicity by using a systematic literature review and meta-analysis.

## 2. Methods

### 2.1. Literature Search Strategy

A systematic search was carried out by two investigators independently using PubMed, Web of Science, and EMBASE to find studies published before 26 January 2022. The following search terms were included: (cisplatin OR oxaliplatin OR carboplatin OR platinum compounds) AND (glutathione transferase* OR GST OR GSTM* OR GSTT* OR GSTP* OR GSTP1 OR rs1695 OR Ile105Val) AND (polymorph* OR genotyp* OR null OR deletion OR variant* OR mutation*) AND (toxicity OR adverse OR side-effects OR adverse effects). An initial screening of the titles and abstracts was conducted following the removal of duplicates. The studies that were selected for inclusion in this study were then reviewed in the full text following the eligibility criteria. This study is registered at the INPLASY (approval No. INPLASY202230025).

### 2.2. Inclusion and Exclusion Criteria

Studies were included if they (1) were randomized controlled trials (RCT) or cohort studies; (2) included adult patients receiving platinum-based regimens; (3) evaluated the association of rs1695 SNP with toxicity; (4) included applicable data on genotype in both cases and controls; or (5) published in English. Studies were excluded if they were (1) not involving toxicity outcomes; (2) not involving rs1695; (3) pharmacokinetic studies; (4) reviews, meeting abstracts, case reports, or case series, comments, letters, updates, news, editorials, or conferences; or (5) unable to provide appropriate data.

### 2.3. Data Extraction and Quality Assessment

For each study, the following data were collected: the last name of the first author, the year of publication, the number of patients, the country, the mean and range of the participants’ ages, the percentage of females, the type of cancer, the treatment regimen, and the definitions of toxicity outcomes. Selected studies were evaluated based on the Newcastle–Ottawa Scale (NOS). Among the components of the NOS are subject selection, comparability of study groups, and the exposure or outcome; every study can attain a total score of 9.

### 2.4. Statistical Analysis

To examine the association between the outcomes and polymorphism, odds ratios (ORs) with 95% confidence intervals (CIs) were calculated. The heterogeneity of the selected studies was evaluated using I^2^. If I^2^ was greater than 50%, this indicated a high degree of heterogeneity; we applied the random-effects model. A fixed-effects model was applied when I2 was less or equal to 50%. We considered *p*-values lower than 0.05 to be significant. We performed statistical analyses using Review Manager (RevMan) version 5.4 (The Cochrane Collaboration, Copenhagen, Denmark).

Egger’s and Begg’s regression tests of the funnel plot were conducted to identify publication bias. The analysis of publication bias was conducted using the RStudio software (version 4.0.0; RStudio: Integrated Development for R, Boston, MA, USA). This meta-analysis was written based on the Preferred Reporting Items for Systematic Reviews and Meta-Analyses (PRISMA) 2020 Statement which comprises a checklist of 27 items.

## 3. Results and Discussion

**Figure 1:**
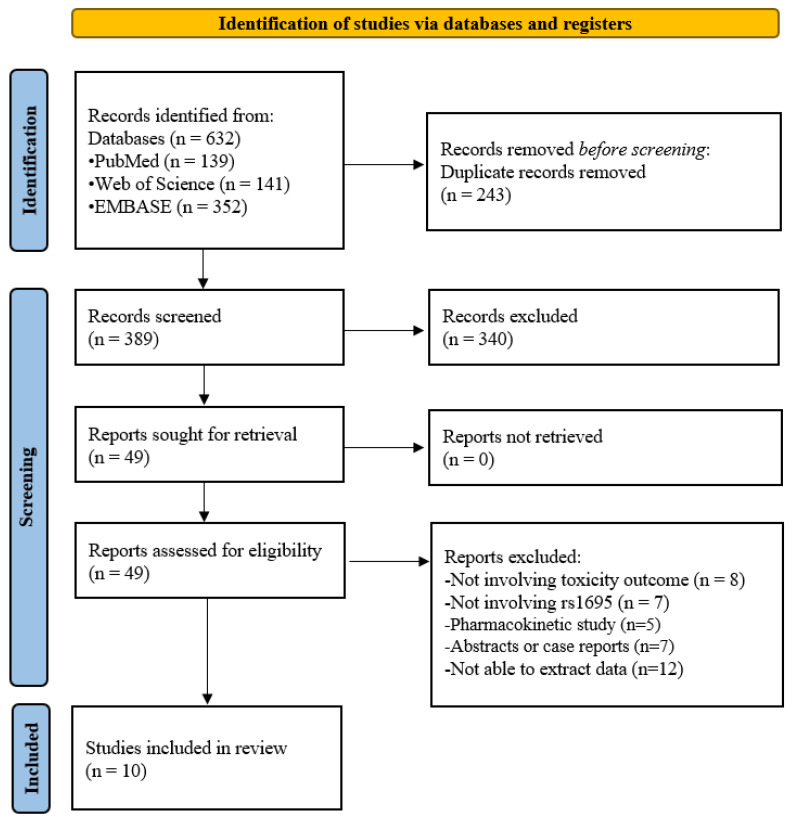
Flow diagram of the study selection process.

Figure 1 shows a flow diagram of the literature search and selection process. There were a total of 632 records initially searched from PubMed (*n* = 139), Web of Science (*n* = 141), and EMBASE (*n* = 352). After removing duplicates (*n* = 243) and irrelevant studies from the title and abstract (*n* = 340), 49 records were selected for full-text review. Thirty-nine records were excluded due to the following reasons: not involving toxicity outcome (*n* = 8), not involving rs1695 (*n* = 7), pharmacokinetic studies (*n* = 5), abstracts, or case reports (*n* = 7), or unable to extract data (*n* = 12). Therefore, this meta-analysis included ten studies [3,7,8,11,12,13,14,15,16,17].

Table 1 shows the baseline characteristics of the included studies. The studies were published between 2006 and 2021. Six studies were performed in Asia, two in Europe, and two in South America. This study included patients with different types of cancer, including esophageal cancer, lung cancer, and colon cancer. The studies with platinum-based regimens, including cisplatin, oxaliplatin, or carboplatin, were evaluated. Using NOS, each study received six or higher than six points out of nine.

**Figure 2:**
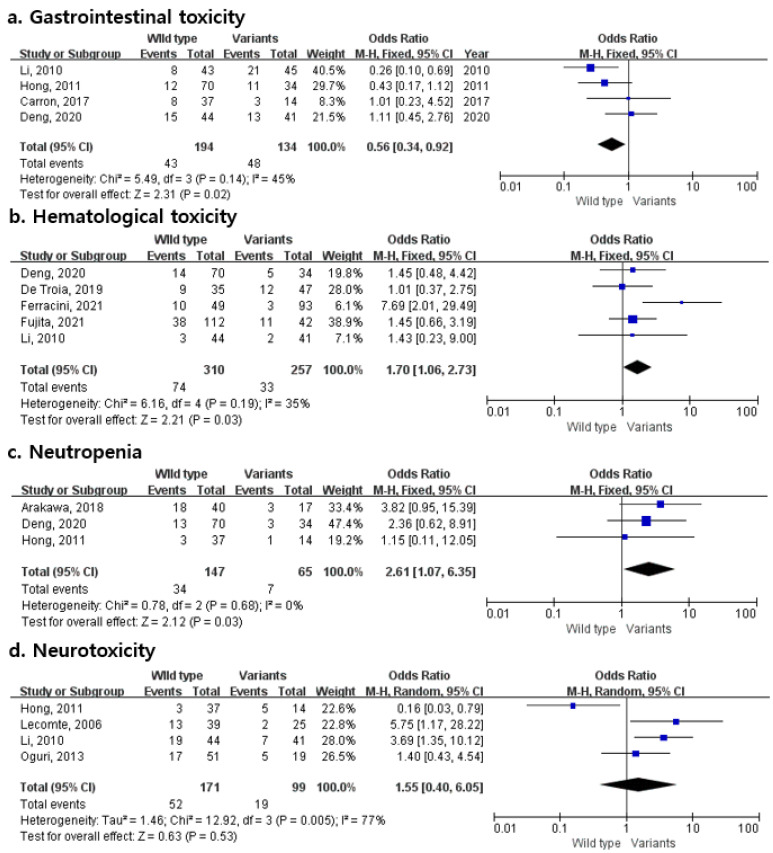
Forest plots of the association between platinum-induced toxicities and *GSTP1* polymorphism (**a**) gastrointestinal toxicity; (**b**) hematological toxicity; (**c**) neutropenia; (**d**) neurotoxicity.

Figure 2 presents the OR and 95% CIs relating to the rs1695 polymorphism in the GSTP1 gene with the risks of various platinum-associated toxicities, such as gastrointestinal toxicity, hematological toxicity, neutropenia, and neurotoxicity. Gastrointestinal toxicity involved four studies and suggested that the rs1695 polymorphism was associated with an approximately 44% decrease in the odds of toxicity compared to the wild-type homozygotes. There were five hematological toxicity and three neutropenia studies in which the rs1695 polymorphism was associated with elevated toxicities by about 1.7- and 2.6-times, respectively (95% CI 1.06–2.73 and 1.07–6.35, respectively).

Since there was no heterogeneity among the included studies for gastrointestinal toxicity, hematological toxicity, and neutropenia (I^2^ = 45%, 35%, and 0%, respectively), the effect size was calculated using a fixed-effects model. A random-effects model was applied to neurotoxicity (I^2^ = 77%). Egger’s and Begg’s tests did not show evidence for publication bias in this meta-analysis (gastrointestinal toxicity: Egger’s test, *p* = 0.776; Begg’s test, *p* = 0.500; hematological toxicity: Egger’s test, *p* = 0.520; Begg’s test, *p* = 0.142; neutropenia: Egger’s test, *p* = 0.430; Begg’s test, *p* = 0.602; neurotoxicity: Egger’s test, *p* = 0.536; Begg’s test, *p* = 0.174, respectively).

The principal finding of this study is that patients with the G allele of rs1695 have an approximately 1.7% and 2.61% increased odds of platinum-induced hematological toxicity and neutropenia, respectively (95% CI 1.06–2.73 and 1.07–6.35). On the other hand, the rs1695 polymorphism resulted in a 44% decrease in the odds of gastrointestinal toxicity compared to the wild-type homozygotes. The Begg’s and Egger’s tests showed no significant publication bias for toxicities.

Platinum-containing chemotherapy is the largest class of drugs used for cancer treatment and exhibits excellent drug responses. Identical DNA adducts formed by these anti-cancer medications [18] induce various cellular responses, such as transcriptional inhibition, cell cycle arrest, and DNA repair [19,20]. Together, platinum-based anticancer drugs inhibit tumor cell transcription and cause cell cycle arrest via the created DNA adducts. Moreover, platinum-based chemotherapy is advantageous because the platinum complex structure is designed to optimize DNA interaction. The enhanced permeability and retention effect has been applied to the development of the medications [21]. Yet, despite their clinical efficacy, platinum-based anti-cancer medications also exhibit various adverse events.

By catalyzing mercapturic acid synthesis, GSTs act as the first step in eliminating toxic compounds [22]. GSTP1, belonging to a class of GSTs, is known as one of the most vital detoxification enzymes in the body. It is responsible for initiating a pathway to lower the intracellular concentration of platinum medications and eliminate toxic compounds [23]. As GSTP1 polymorphisms can cause GST abnormalities, genetic testing can be suggested for patients receiving platinum-based chemotherapy. While several genetic differences in the GSTP1 gene are hypothesized to affect GST function, research is still ongoing to identify specific variants associated with platinum-induced toxicity.

Several studies have revealed that the rs1695 of GSTP1 mutation results from an A to G change at codon 105, causing a transition from isoleucine to valine, which is associated with decreased enzymatic activity and greatly increased platinum-related toxicities [24,25,26]. Previous studies have investigated the association between the GSTP1 rs1695 gene polymorphism and platinum-induced toxicities [24,27,28]; however, the conclusions were not consistent. Moreover, most meta-analyses were focused on one specific outcome, which created a need to employ a meta-analysis to amalgamate the various outcomes with a larger number of subjects and updated records.

This study showed that patients receiving platinum-based treatment with the G allele of rs1695 had about 1.7- and 2.6-fold higher hematological adverse events and neutropenia compared to those with the AA genotype, respectively. Hematological toxicity and neutropenia are serious adverse events that lead to treatment discontinuation. In this context, results from this study indicated that GSTP1 might serve as a potential marker and influence treatment regimens substantially.

Interestingly, unlike hematological adverse events and neutropenia, contrary results were found regarding gastrointestinal toxicities; the G allele carriers had an approximately 44% decrease in the odds of gastrointestinal toxicities compared to patients with the AA genotype. This may be attributable to the tissue-specific expression of rs1695. An expression quantitative trait loci (eQTL) analysis showed that the minor allele of rs1695 was associated with the decreased expression of GSTP1 in the colon and esophagus (gastroesophageal junction); therefore, lower GSTP1 expression could be related to decreased gastrointestinal toxicities. Although gastrointestinal adverse events are the most common toxicities from platinum-based regimens, previous studies failed to show significant associations or consistent results. This study provided a precise estimate of the effects by combining studies to increase the sample size and power. Thus, determining an individualized treatment strategy based on the polymorphism and estimating the patient’s risk of such toxicities would be important aspects in real clinical settings.

This meta-analysis has several limitations. First, the occurrence of platinum-induced toxicities can be affected by various factors, including other co-medications. Due to the inability to collect raw data, this study could not adjust for confounding factors when pooling odds ratios. Second, due to limited data availability, other critical toxicities including nephrotoxicity or cardiotoxicity could not be analyzed. Third, despite the lack of evidence of publication bias, the possibility cannot be ruled out because of the small sample size.

## 4. Conclusions

This meta-analysis identified an association between the selected SNP and platinum-induced complications by improving the estimation of its effect size. In particular, this study also demonstrated that rs1695 expression was tissue-specific and had opposite effects in different tissues. Based on this systematic review and meta-analysis on the rs1695 polymorphism and platinum-related toxicity, the future clinical decision regarding platinum-based regimens can be made.

## Figures and Tables

**Table 1:** Characteristics of studies included.

Authors	Number of Patients	Country	Age (Range, Years)	Female	Cancer Type	Treatment Regimen	Definition of Outcome	Total NOS
Arakawa, 2018 [8]	57	Japan	66 (45–77)	11%	Esophageal squamous cell carcinoma	Cisplatin-based chemotherapy	CTCAE v4.0	7
Carron, 2017 [7]	88	Brazil	54 (27–66)	8%	Head and Neck Cancer	Cisplatin-based chemotherapy	NCI criteria v4.0	6
De Troia, 2019 [12]	82	Italy	Not indicated	37%	Lung cancer	Platinum-based chemotherapy	CTCAE v4.03	6
Deng, 2020 [13]	104	China	56 (25–78)	47%	Colorectal cancer	Oxaliplatin-based chemotherapy	NCI CTCAE v3.0	8
Ferracini, 2021 [14]	112	Brazil	58 (22–87)	100%	Ovarian Cancer	Carboplatin-based chemotherapy	CTCAE v5.0	8
Fujita, 2021 [15]	239	Japan	64.0 (41–83)	13%	Esophageal cancer	Platinum-based chemotherapy	CTCAE v5.0	7
Hong, 2011 [11]	52	Korea	63.0 (37–74)	29%	Metastatic colorectal cancer	Oxaliplatin-based chemotherapy	NCI CTC v3.0	8
Lecomte, 2006 [3]	64	France	64 (24–84)	45%	Gastrointestinal solid tumors	Oxaliplatin-based chemotherapy	Not indicated	6
Li, 2010 [16]	89	China	55 (32–77)	28%	Advanced Gastric Cancer	Oxaliplatin-based chemotherapy	NCI CTC v2.0	7
Oguri, 2013 [17]	70	Japan	65 (37–81)	30%	Colorectal cancer	Oxaliplatin-based chemotherapy	NCI CTCAE v3.0	6

CTCAE: Common Terminology Criteria for Adverse Event; NCI: National Cancer Institute; CTC: Common Toxicity Criteria; NOS: Newcastle-Ottawa scale.

## Data Availability

Data sharing not applicable.
